# Oligomeric Forms of Insulin Amyloid Aggregation Disrupt Outgrowth and Complexity of Neuron-Like PC12 Cells

**DOI:** 10.1371/journal.pone.0041344

**Published:** 2012-07-27

**Authors:** Ehsan Kachooei, Ali Akbar Moosavi-Movahedi, Fariba Khodagholi, Hassan Ramshini, Fatemeh Shaerzadeh, Nader Sheibani

**Affiliations:** 1 Institute of Biochemistry and Biophysics, University of Tehran, Tehran, Iran; 2 Neuroscience Research Center, Shahid Beheshti University of Medical Sciences, Tehran, Iran; 3 Department of Ophthalmology and Visual Sciences, and Pharmacology, University of Wisconsin School of Medicine and Public Health, Madison, Wisconsin, United States of America; Foundation for Biomedical Research Academy of Athens, Greece

## Abstract

Formation of protein amyloid fibrils consists of a series of intermediates including oligomeric aggregates, proto-fibrillar structures, and finally mature fibrils. Recent studies show higher toxicity for oligomeric and proto-fibrillar intermediates of protein relative to their mature fibrils. Here the kinetic of the insulin amyloid fibrillation was evaluated using a variety of techniques including ThT fluorescence, Congo red absorbance, circular dichroism, and atomic force microscopy (AFM). The solution surface tension changes were attributed to hydrophobic changes in insulin structure and were detected by Du Noüy Ring method. Determination of the surface tension of insulin oligomeric, proto-fibrillar and fibrillar forms indicated that the hydrophobicity of solution is enhanced by the formation of the oligomeric forms of insulin compared to other forms. In order to investigate the toxicity of the different forms of insulin we monitored morphological alterations of the differentiated neuron-like PC12 cells following incubation with native, oligomeric aggregates, proto-fibrillar, and fibrillar forms of insulin. The cell body area, average neurite length, neurite width, number of primary neurites, and percent of bipolar cells and node/primary neurite ratios were used to assess the growth and complexity of PC12 cells exposed to different forms of insulin. We observed that the oligomeric form of insulin impaired the growth and complexity of PC12 cells compared to other forms. Together our data suggest that the lower surface tension of oligomers and their perturbation affects the morphology of PC12 cells, mainly due to their enhanced hydrophobicity and detergent-like structures.

## Introduction

A variety of human diseases including neurodegenerative diseases, non-neuropathic systemic amyloidoses, and non-neuropathic localized diseases are related to formation of protein amyloid fibrillar aggregation [1], [2]. The formation of amyloid fibrils is a generic characteristic of proteins and peptides and, it is not exclusive to proteins that cause diseases [3]–[6]. A large number of proteins aggregate to amyloid fibrils or amyloid-like states under non-biological conditions [3]. During amyloid formation of different proteins, an unfolded or partially unfolded state causes the formation of non-fibrillar aggregation prior to amyloid formation [7]. Amyloid fibril formation consists of a series of stages including soluble oligomer aggregation as a result of non-specific interactions, protofibrillar structure formation and their assembly to mature fibrils [8]–[11]. Insulin prefibrillar aggregations (oligomers and protofibrils) have a low content of beta sheets in comparison with mature amyloid fibrils, and act as a nucleation agent to form mature fibrils [12], [13].

The different aggregate forms of proteins are cytotoxic and can disrupt various biochemical processes including cell membrane and normal ion gradient [14], inactivate other normal, functional proteins and obstruct chaperon proteins [15], initiate membrane permeabilization [16], generate reactive oxygen species (ROS) [17] and dysregulate cytosolic Ca^2+^ [18], induce apoptotic responses [19] and finally cell death [20]. Cytotoxicity of amyloid aggregation formed by proteins, whether associated with diseases or not, is an inherent phenomenon and correlates with their common structure and not the sequence of amino acids [21]. It has been shown that oligomeric intermediates are more toxic species relative to mature fibril forms. Thus, exploring the processes that are involved in the formation of these intermediates is of significant importance [22], [23].

Insulin is a protein hormone which regulates glucose uptake by binding to insulin-receptors in the surface of cell membrane that result in stimulating of signaling pathway in cell [24]. It has a small structure including two helical chains (A and B chains, containing 21 and 30 amino acid residues, respectively), which are linked through two disulfide bonds [25]. Insulin amyloid formation is characteristic of localized amyloidosis that is observed in patients with insulin dependent diabetes who frequently receive insulin [26], [27]. The fibrillated forms of insulin lose therapeutic effectiveness and can trigger immune responses as a result of frequent injections [27], [28]. In vitro, insulin has a tendency to undergo fibrillation under conditions that result in partially unfolded intermediates such as high temperature, low pH, high concentration, and incubation with organic solvents [9], [29]–[32]. Kinetics of insulin fibrillation is like kinetics of other proteins and includes three stages nucleation, elongation and saturation [8]. The cytotoxicity of insulin fibrillar species has been observed in rat pheochromocytoma PC12 cells [28], [33] and pancreatic β-cells [34]. So, in the present work, we used insulin as a model system for studying amyloid fibrillation.

**Figure 1:**
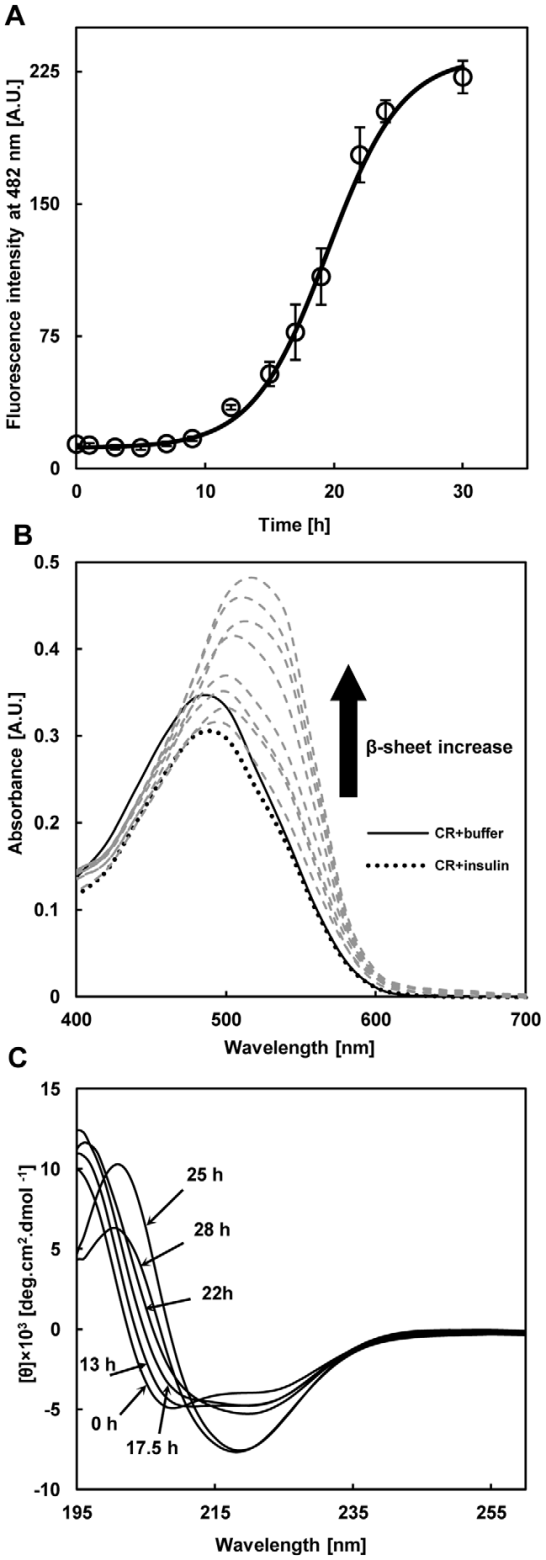
The kinetics of amyloid fibrillation induced by dissolving insulin (0.6 mg/mL) in glycine buffer (20 mM, pH 2.0) and 37°C and agitation. **A**) The content of fibril formation was recorded via ThT fluorescence as a function of incubation time. Data represent the average of 3 independent measurements and error bars represent standard deviation from the mean value. **B**) The content of fibril formation was also recorded via Congo red absorption spectrum during incubation time. Congo red spectrum alone (**^____^**) with native insulin (**….**) and with incubated samples (**- - -**) after 10, 13, 15, 18, 20, 23, 25 and 30 h, respectively. **C**) Far-UV CD spectra of bovine insulin during incubation time. At the onset of incubation (0h) two minima at 208 and 222 nm indicate α–helical structure. After 28 h of incubation, appearance of a new minimum at 216 nm indicates cross β–sheet structure because of amyloid fibrillation.

In the aqueous solution containing protein molecules, the changes in protein concentration, pH, temperature and salt ions, which affect protein structural stability and conformation, alter the surface tension of solutions at the air/water interface [35], [36]. The solution surface tension is an important criterion which correlates with protein conformational stability and aggregation [37]. It has been demonstrated that an increase in surface tension of aqueous solution with small molecules results in stabilization of insulin and inhibits its aggregation [38]. The alteration of surface tension by targeted peptides has also different impact on protein aggregation [27]. Thus, surface tension is a feature of protein hydrophobicity at the interface layer of aqueous solution and is used to determine the conformational changes and stability of proteins [36].

**Figure 2:**
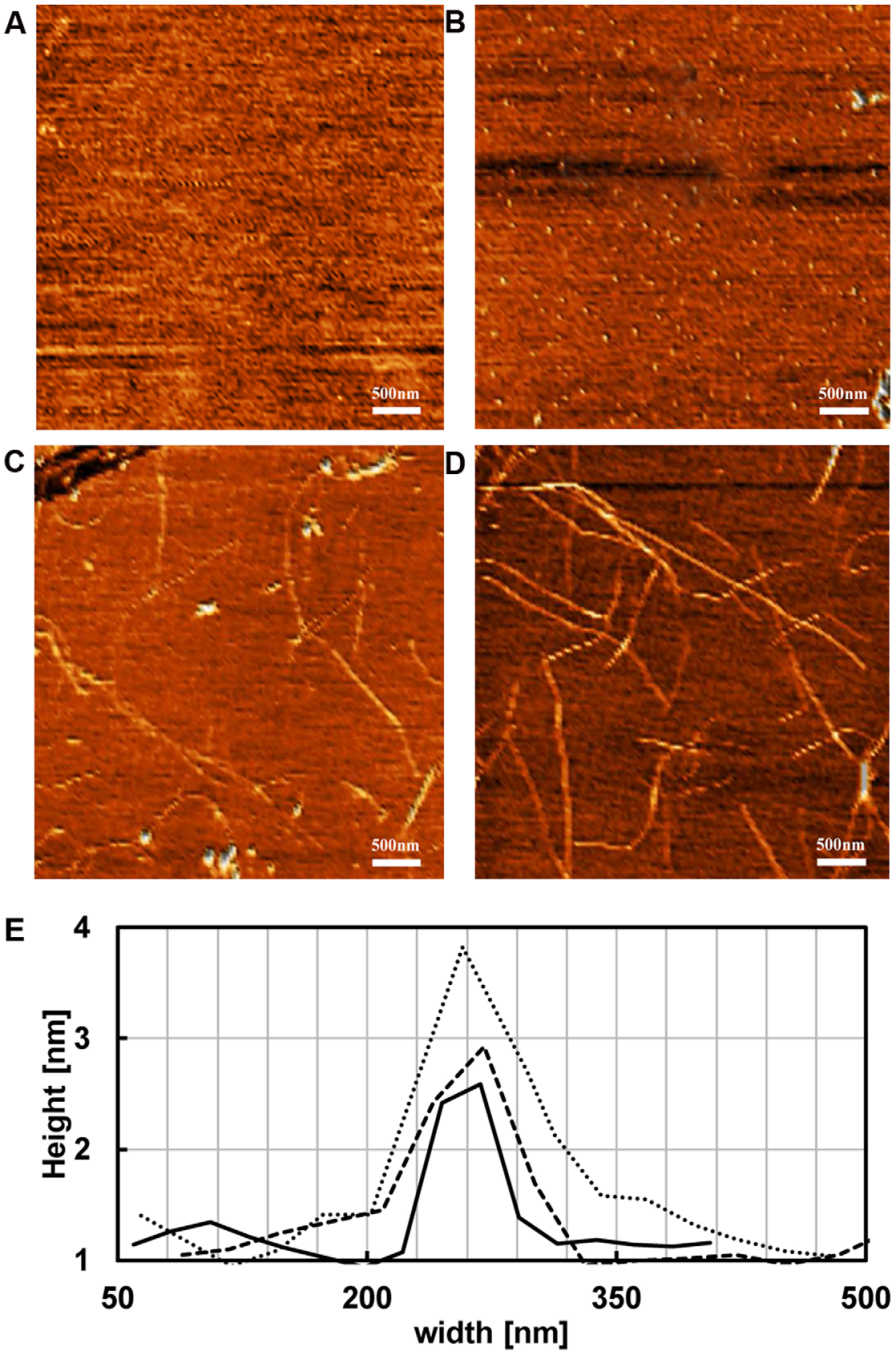
AFM images were recorded at different time points of insulin fibrillation as deducd from ThT fluorescence profile. **A**) 0 h showing no remarkable structure for native insulin, **B**) 12 h, spherical shapes as oligomer structures, **C**) 22 h, single strand proto-fibrils, **D**) 30 h, long and mature fibrils with visible nodes because of twisted proto-fibrils. The scale bar represents 500 nm.

**Figure 3:**
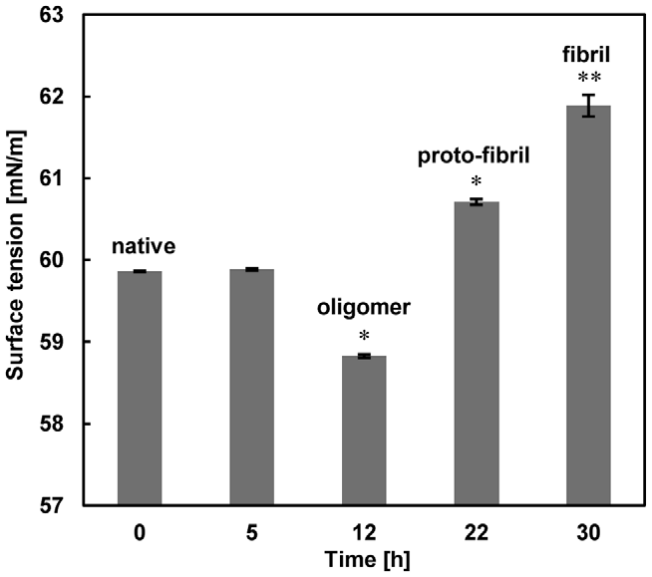
Surface tension changes for various forms of insulin showing the extent of surface tension of solutions containing insulin with native, oligomer intermediates, proto-fibril, and fibril structures at the air-water interface. Data represent the average of decuple measurements and error bars represent standard deviation (SD). *Significantly different from native insulin. Statistical significances were achieved when p<0.05.

In the living cells, morphology has an important role in cellular function especially in specialized cells such as neurons, which is referred to as “function follow form” principle. In the nervous system, there is a strong relation between the specific shape of neurons and their characteristic activity [39], [40]. As a result, any changes in the neuron form and its branching patterns can affect its function. Several factors can affect the shape of a neuron.

In this paper, we attempted to investigate the toxicity of the different forms of insulin which monitored the morphological alterations of the differentiated neuron-like PC12 cells following incubation with native, oligomeric aggregates, proto-fibrillar, and fibrillar forms of insulin.

## Materials and Methods

### Materials

Bovine pancreas Insulin (code I-5500), Thioflavin T, Congo red, Dulbecco’s modified Eagle’s medium (DMEM) and nerve growth factor (NGF) were from Sigma-Aldrich (USA). Horse serum and fetal bovine serum were from Gibco (USA). Glycine, sodium phosphate, potassium phosphate and sodium chloride were from Merck (Germany).

**Figure 4:**
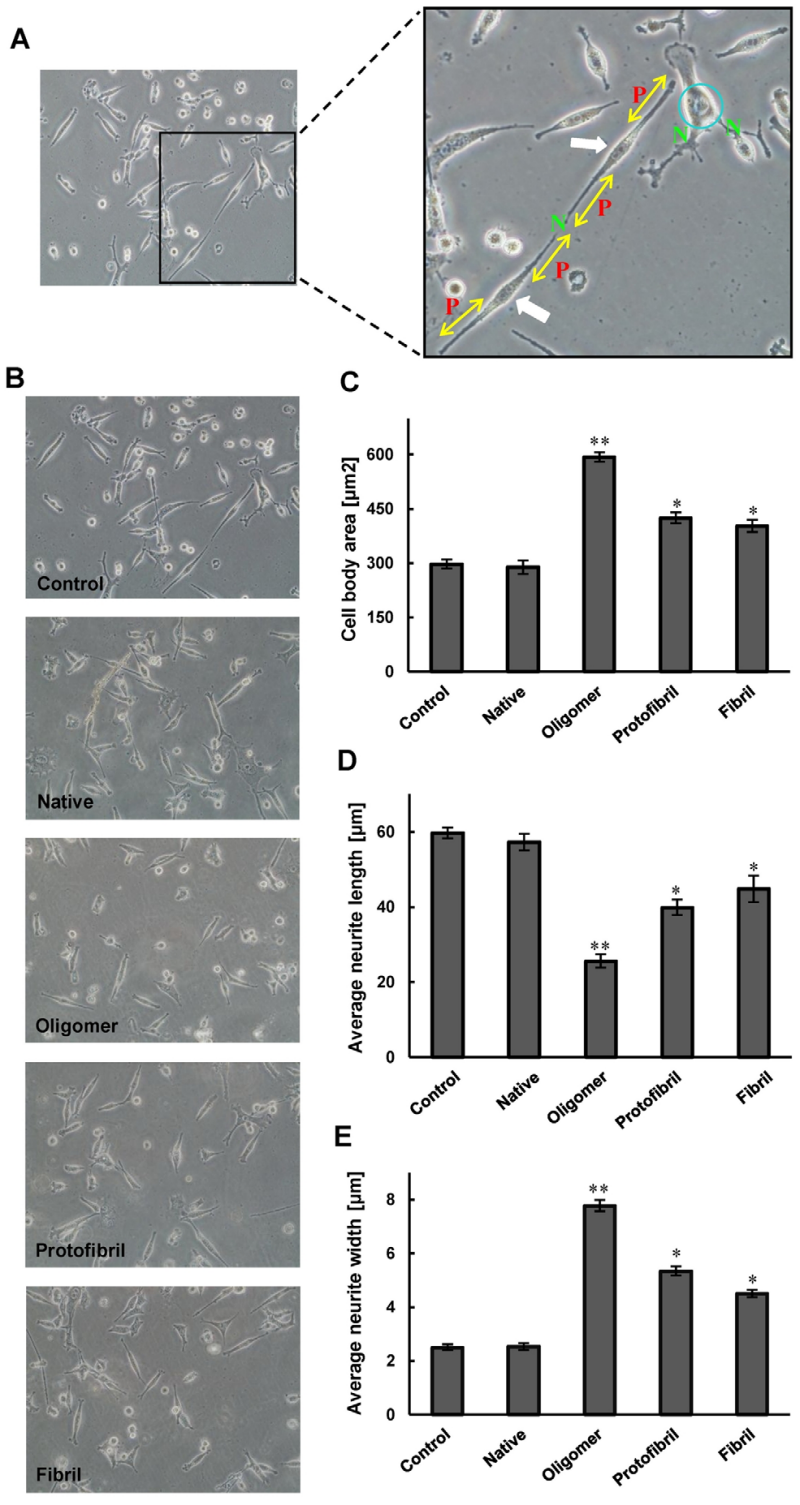
The effects of oligomer, proto-fibril and fibril forms of insulin on neurite outgrowth in differentiated PC12 cells. **A**) The criteria of PC12 differentiation are shown on three neurons (left image) of a sample image. The “P” on right image indicates the primary neuritis of a neuron. The yellow arrow shows the length of a neurite, extent elongated, and membrane-enclosed protrusions of cytoplasm. The blue circle on right image shows the cell body. Neurite width is not equal in all parts of neurons, thus the average neurite width must be calculated by dividing cell body area to average neurite length. The white arrows show to bipolar cells. The letter “N” indicates the nodes, the sites at which individual neurites branched or separate neurites contacted each other. The criteria were quantified 12 h after treatment; **B**) NGF-differentiated PC12 cells were pretreated with three amyloid intermediate forms of insulin. C) Cell body area; D) average neurite length; and E) average neurite width. *Significantly different from control cells. Statistical significances were achieved when p<0.05 (*p<0.05 and **p<0.01).

### Insulin Amyloid Fibrillation

Insulin samples were prepared at a concentration of 0.6 mg/mL by dissolving in glycine buffer (20 mM, pH 2.0) and were incubated at 37°C, accompanied with agitation to induce fibrillation. The insulin solution concentration was determined using Shimadzu UV-Vis-NIR recording spectrophotometer (model UV-3100, Japan)at 276 nm with an extinction coefficient of 1.0 for 1.0 mg/mL [37].

**Figure 5:**
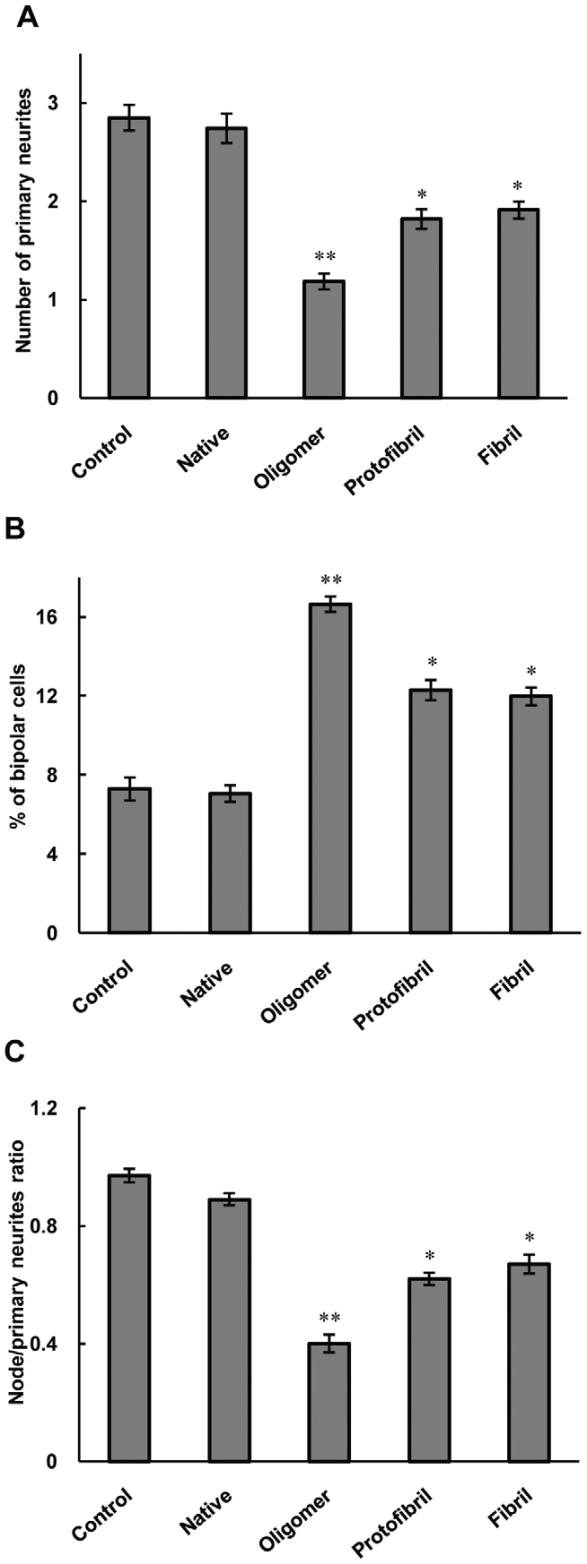
The effects of oligomer, proto-fibril and fibril forms of insulin on neurite complexity in differentiated PC12 cells. The criteria were quantified 12 h after treatment; **A**) primary neurites per cell; **B**) percent of bipolar cells and **C**) the ratio of nodes to primary neurites. * Significantly different from control cells. Statistical significances were achieved when p<0.05 (*p<0.05 and **p<0.01).

### ThT Assay

Stock solution of ThT was prepared in phosphate buffer (25 mM, pH 6) at a concentration of 2.5 mM and stored at 4°C. Concentration of ThT was determined with a molar extinction coefficient of 26,600 M^−1^ cm^−1^ at 416 nm using a spectrophotometer [27]. At different time intervals, an aliquot (10 µL) of incubated solution was mixed with 490 µl of ThT solution (25 µM) in a quartz cuvette with 1 cm path length. We used a Cary Eclipse fluorescence spectrophotometer (Varian, Australia) for fluorescence intensity measurements. The samples were excited at a wavelength of 440 nm, and emission was detected at 482 nm. The excitation and emission slit width were set at 5 and 10 nm, respectively. The acquired data from ThT fluorescence measurements were fitted to the sigmoid curve depicted by the following equation [32]:


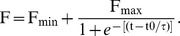


where F is fluorescence intensity at time t, F_min_ and F_max_ represent fluorescence intensity at initial time and saturation phase of incubation, respectively. The t is the incubation time and t_0_ is the time to obtain 50% of maximal fluorescence. The value of τ was obtained by nonlinear regression, apparent rate constant and lag phase time was determined to be 1/τ and t_0_ - 2τ, respectively. Each measurement was done triplet and the averages were used.

### Congo Red Assay

At each time point, 24 µL aliquot of sample was mixed with 176 µL of CR solution (20 µM), prepared in 5 mM potassium phosphate, 150 mM NaCl and pH 7.4. After at least 15 min incubation at room temperature, absorption spectrums were recorded from 400 nm to 700 nm using a UV-Vis-NIR recording spectrophotometer (model UV-3100, Shimadzu, Japan) and a quartz cuvette with 1 cm path-length.

### Circular Dichroism Spectroscopy

Far-UV CD was used for analyzing secondary structure during fibrillation. We diluted 50 µL of sample solution 2-fold in glycine buffer (20 mM, pH 2.0) in a quartz cell with a 1 mm path length. The CD spectra measurement was recorded on a circular dichroism spectrometer (model 215, Aviv, USA) by scanning the sample from 195 nm to 260 nm at 25°C using a bandwidth of 1 nm, a step interval of 1 nm, an average time of 0.5 s, and a slitwidth of 0.02 mm. The spectrum of glycine buffer was subtracted from sample spectra for data analysis.

### Atomic force Microscopy (AFM)

In order to visualize insulin amyloid fibrillation the atomic force microscopy was used. For this purpose, an aliquot (10 µL) from the incubated solution was placed on freshly cleaved mica at room temperature. After a few minutes mica was slowly washed with 100 µL of deionized water and followed by drying with nitrogen gas. Each image was acquired in a tapping mode at a scan speed of 30 µm/s, loop filter of 3 Hz and force of 200 nN with a Dual Scope Probe Scanner (model DS 95-50-E, DME, Denmark) with an area of 5×5 µm^2^. Conical shape silicon tips (mikromasch NSC 16) with a resonance frequency of 150 kHz and a nominal constant of 40 N/m were used.

### Surface Tension Measurement

The solution surface tension was measured with a Krüss K100 tensiometer using Du Noüy Ring method at 25°C. For each measurement, sample solutions were diluted to a concentration of 0.2 mg/mL and in a volume of at least 2 mL. Surface tension detection was used at appropriate time of incubation according to the ThT fluorescence kinetic profile for each intermediate (native insulin, oligomer, proto-fibril and fibril). The measurements were done for each intermediate and the average was used for data analysis.

### Cell Culture and PC12 Cell Differentiation

Rat pheochromocytoma (PC12) cells were obtained from Pasteur Institute (Tehran, Iran) which were grown in DMEM, supplemented with 5% fetal bovine serum, 10% horse serum, and 1% antibiotic mixture comprising penicillin-streptomycin, in a humidified atmosphere at 37°C with 5% CO_2_. Growth medium was changed three times a week. The cells were differentiated by incubating with nerve growth factor (NGF; 50 ng/mL) every other day for 6 days.

### Treatment Conditions and Morphological Analysis of Differentiated PC12 Cells

Differentiated PC12 cells, seeded in 6-wells plates, were incubated with 16 µM concentration of Insulin and its three amyloid intermediates (Oligomer, Protofibril and Fibril) for 12 h. For morphological analysis, two random images were acquired from each well by phase contrast microscopy (Olympus, IX71). A minimum of 50 cells per treatment were quantified. The cell body was the criteria for selection. Processes were completely within the field of view, and the cell body of individual cell was distinct from neighboring cell bodies. Cells fitting these criteria were analyzed and their cell body area, average neurite length, neurite width, and the number of primary neurites and bipolar morphology were quantified. Cell body area was defined as the area of the cell that neurite processes originate from and contains cell organelles. Neurite length was calculated by summing the lengths of the primary process and all associated branches. To establish the average neurite width, the outlines of individual primary neurites were traced. The cell body area calculated by Cell A software and then divided by the length of the neurite. Primary neurites were defined as clear protrusions from the cell body that are greater than 10 µm in length. Cells were considered “bipolar” if they displayed a cell body with one process at each end. To evaluate neurite networks, images were analyzed using the cell counter plug-in to score all branching nodes in each image. Nodes were defined as sites at which individual neurites branch or separate neurites contacted each other.

All measurements expressed as proportions that used the number of cells displaying the characteristic of a sub-population of the total number of cells which met the selection criteria described above.

### Data Analysis

All data are represented as the mean ± S.E.M. Comparison between groups was made by one-way analysis of variance (ANOVA) followed by a specific post-hoc test to analyze the difference. The statistical significances were achieved when P<0.05 (*P<0.05, **P<0.01 and ***P<0.001).

## Results and Discussion

The aim of the present study is measurement of surface tension of the solutions containing various forms of insulin (native, oligomer, proto-fibril and fibril) and characterizing the surface properties of these forms. Also, since aggregation of proteins can disrupt biochemical processes and signal transduction in these cells, here we investigated the effect of insulin and its three amyloid intermediates (oligomer, proto-fibril and fibril forms) on the differentiation and morphology of PC12 cells (as a model for neuronal cells). This finding is the correlation between surface properties of assembled forms of insulin and their disrupting effects on cells.

### Kinetics of Insulin Fibrillation Utilizing ThT and Congo Red

Thioflavin T and Congo red were used as markers for detection of amyloid fibrillation because of their binding tendency to cross beta sheet structures (a key feature of amyloid fibrils) [41]. Fig. 1A shows the maximum emission of ThT fluorescence emission at 482 nm. At the initiation time of insulin incubation at acidic pH and 37°C, ThT fluorescence intensity was low and after approximately 30 h reached maximum. The apparent rate constant for amyloid fibrillation was found to be 0.345 h^−1^ and the lag time was determined to be 13.7 h. We also utilized Congo red binding as a complementary evaluation of amyloid fibrillation. Fig. 1B shows during incubation of insulin for 30 h, an increase in intensity of Congo red with more red shift was observed relative to Congo red spectra alone or native insulin. Thus, there is an increase in intensity at 540 nm that is a characteristic of more Congo red binding to amyloid fibrils [41].

### Secondary Structure Transition During Incubation

Native insulin has a CD spectrum with two minima at 208 and 222 nm that are characteristic of dominant α-helical structure [9]. In this study, the CD spectra of insulin samples at the initiation of incubation (0 h), verified the presence of the two minima at 208 and 222 nm for native insulin (Fig. 1C). During the incubation at low pH at 37°C, the two minima disappeared slowly indicating a decrease in α-helical structure followed by the appearance of a single minimum at 216 nm indicating a dominantly β-sheet structure. After 28 h of incubation the intensity of this minimum reached a higher value indicating more β-sheet structure.

### Morphology Analysis of Insulin Fibrillation

To analyze the insulin morphological characteristics during fibrillation, we used sample aliquots at the initiation, end of lag phase, end of elongation phase, and saturation phase, based on ThT fluorescence profiles. As shown in AFM images (Figs. 2A-D), at the initiation of incubation no remarkable structure was observable on the mica surfaces (Fig. 2A). At the end of lag phase, spherical bead-like shapes by ∼60 nm width became visible, which indicate oligomer structure formation (Fig. 2B). Thus following incubation at the end of elongation phase, single strands and unbranched fibrils were formed (∼100 nm thickness) which were distinct as proto-fibrillar (or proto-filament) structures (Fig. 2C). The following were finally observed in saturation phase: 1) long and straight mature fibrils by ∼150 nm thickness which were thicker than proto-fibrils, and 2) showing distinct nodes that suggest intertwined proto-fibrils (Fig. 2D) [11]. Fig. 2E shows section profiles of oligomer, proto-fibril and fibril structures. The thickness and height of each one are shown in this figure.

### Oligomeric Forms of Insulin Decreased the Surface Tension of the Solution

According to AFM images and ThT fluorescence profiles, we measured surface tension of insulin sample solutions at the air-water interface, for native, oligomer aggregates, proto-fibril and mature fibril forms at a concentration of 0.2 mg/mL. Proteins are highly surface active and easily adsorb at the air-water interface. Any change in physicochemical properties of proteins such as conformational changes, results in alteration of surface properties such as surface tension of protein solution [42]. The amphiphilic structure of proteins, which is the result of their hydrophilic and hydrophobic side chains, cause them to be adsorbed at the air-water interface [43]. Fig. 3 shows that the surface tension of native insulin was approximately 59.8 mN/m and after 5 h of incubation, surface tension of solution was not significantly changed in comparison with native insulin solution. However a significant decrease in surface tension of solution, after 12 h of incubation, for oligomer sample was noted (∼58.8 mN/m). This decrease in surface tension was accompanied with the conformational changes in insulin.

Surface tension is used for measurement of the hydrophobicity of adsorbed proteins at the air-water interface [36], [44]. The decrease in surface tension of oligomeric forms represents an increase in hydrophobic content of the structure. Conformational changes and hydrophobic interactions are the driving forces for faster adsorption of oligomers at the air-water interface compared to the native form of insulin, and subsequently lowering the surface tension. Partial unfolding and exposure of hydrophobic protein surfaces result in intermolecular interactions between denatured protein molecules and finally their aggregation [45]. Due to exposure of hydrophobic surfaces and larger surface area, oligomer structures have a more extensive hydration shell than native insulin molecules [46]. The surface tension for proto-fibril solution showed an increase relative to both oligomer and native insulin solution. The surface tensions for proto-fibril and fibril solutions were approximately 60.7 and 61.8 mN/m, respectively.

Increased surface tension, followed by the formation of proto-fibril and fibril structures, implies reduced hydrophobic protein surfaces which are replaced with hydrophilic ones. These results confirmed that formation of proto-fibril and fibril, and accordingly increased surface tension, were accompanied with a thermodynamic driving force to diminish surface area and interfacial energy that result in more stable structure of insulin with lower energy state [37], [47]. Based on the experimental condition of this study (pH 2.0, 37°C and agitation), the monomeric insulin became partially unfolded or unstructured, and the hydrophobic surfaces were exposed to solution molecules. This state is thermodynamically unstable, and fibrillation occurs to form a self-assembled state of insulin to stabilize it [27]. Oligomeric structures are formed, as the result of intermolecular hydrophobic interactions, following more hydrophobic structures with larger surface areas. By formation of proto-fibrils and fibrils, hydrophobic surfaces become buried in the core of these structures and are replaced with hydrophilic surfaces. Thus, surface area and hydration shell are diminished to form a more compact structure and subsequently increased surface tension of solution.

### Oligomer, Proto-fibril and Fibril Forms of Insulin Impaired Neurite Outgrowth in Differentiated PC12 Cells

Three criteria, cell body area, average neurite length and average neurite width, were selected to monitor cell growth. As shown in Figs. 4B and C, average cell body area increased in oligomer, proto-fibril and fibril insulin exposed cells, compared to control and native neurons. Effects of these structures on neurite length contrasted with the results for cell body area (Fig. 4D). Moreover, neurites exposed to these forms of insulin were not shorter, but were even wider than those in control cultures. Because of variability along the length of the neurite, the total area of the neurite was divided by the neurite length to calculate the average neurite width. This ratio was significantly higher in cells treated with oligomer, proto-fibril and fibril forms of insulin after 12 h (Fig. 4E). Furthermore, for each criterion, the effect of oligomer form of insulin was more prominent than the proto-fibril form and the proto-fibril form was more significant than the fibril form.

### Oligomer, Proto-fibril and Fibril Forms of Insulin Decreased Neurite Complexity in Differentiated PC12 Cells

Specific parameters of morphological complexity were also measured. First, the number of primary neurites (>10 µm) emanating from individual cell bodies was measured. Fig. 5A shows the number of primary neurites per cell body, which decreased in insulin oligomer, proto-fibril and fibril forms treated cells. In contrast, the proportion of cells with the very simple bipolar morphology of a cell body and only two neurites increased in cells treated with these forms of insulin, compared to control and native cultures (Fig. 5B). As the final parameter of complexity, we calculated the ratio of total neurite branching nodes to total number of primary neuritis. Fig. 5C shows the ratio of nodes to primary neuritis which decreased in cells treated with oligomer, proto-fibril and fibril forms of insulin, whereas the ratio was significantly increased in the native structure. Thus, the three amyloid intermediate forms of insulin decreased the complexity of PC12 cells.

The increase in number of bipolar cells exposed to the three forms of insulin was in agreement with the reduction of complexity of these cells. Several lines of studies indicated that reduction of dendrite structure and neuronal complexity are associated with disruption of neuronal function [48], [49]. Thus, the three intermediate structures of insulin including oligomer, proto-fibril and fibril forms can affect the function of PC12 cells via decreasing neuronal dendritic branches. The accumulation of Aβ and tau-induced changes are shown to be pathological hallmarks of Alzheimer Disease, and are believed to contribute to many of the alterations in neuronal structures [50]. Thus, the oligomeric structure of insulin has characteristic similar to Aβ here. In our experiments, differentiated PC12 cells were used since they are well characterized and exhibit unique sensitivity to neurotoxicity. They have been widely used as an experimental model for this purpose [51], [52].

The current study provides, to the best of our knowledge, the first detailed analysis of the effects of different structural forms of insulin on neuronal morphology. However, the intracellular mechanism of this effect is not clear and needs to be further studied.

### Conclusions

The studies presented here indicate that there is a correlation between surface tension and neurotoxicity of various aggregated species in the course of insulin fibrillation. Decreased surface tension, when oligomeric aggregates form, was accompanied with increased neurotoxic effects of these forms. In the case of proto-fibrils and mature fibrils, the increasing surface tension was accompanied with decreased neurotoxic effects. Thus, the quantity of surface tension is an indicator of the intensity of the neurotoxic effects of aggregated species. Oligomeric early aggregates are disorganized structures which expose to the outside hydrophobic surfaces of the protein that are normally buried in the core of globular state [53]. Amphiphilic, detergent-like structure and hydrophobicity of oligomers provide them the capacity to adsorb at the air-water interface, subsequently causing a decrease in surface tension. Moreover, due to hydrophobicity and by a nonspecific detergent-like mechanism, oligomers interact with membranes [18], [54], trigger destabilization and permeabilizition that can be the reason for toxic responses of neuron-like PC12 cells and subsequent morphological alterations. Detergent-like characteristic of aggregates, their effect on the surface tension of solution and perturbation features on morphology of neuron-like PC12 cells, diminished by formation of proto-fibrils and mature fibrils. Thus, here, formation of mature fibrils and lower relative neurotoxicity than their oligomeric early aggregates is a protective mechanism [55].
